# Intracellular lipophilic network transformation induced by protease-specific endocytosis of fluorescent Au nanoclusters

**DOI:** 10.1186/s40580-023-00376-4

**Published:** 2023-06-09

**Authors:** Minhee Ku, Jaemoon Yang

**Affiliations:** 1 Department of Radiology, College of Medicine, Yonsei University, Seoul, 03722 Republic of Korea; 2 Systems Molecular Radiology at Yonsei (SysMolRaY), Seoul, 03722 Republic of Korea; 3 Imaging of MechanoBiology (iMechBio) at Yonsei, Seoul, 03722 Republic of Korea; 4 Convergence Research Center for Systems Molecular Radiological Science, Yonsei University, Seoul, 03722 Republic of Korea

**Keywords:** Au nanocluster, Network analysis, Endocytosis, Lipophilics, MT-MMP, Single-cell

## Abstract

The understanding of the endocytosis process of internalized nanomedicines through membrane biomarker is essential for the development of molecular-specific nanomedicines. In various recent reports, the metalloproteases have been identified as important markers during the metastasis of cancer cells. In particular, MT1-MMP has provoked concern due to its protease activity in the degradation of the extracellular matrix adjacent to tumors. Thus, in the current work, we have applied fluorescent Au nanoclusters which present strong resistance to chemical quenching to the investigation of MT1-MMP-mediated endocytosis. We synthesized protein-based Au nanocluster (PAuNC) and MT1-MMP-specific peptide was conjugated with PAuNC (pPAuNC) for monitoring protease-mediated endocytosis. The fluorescence capacity of pPAuNC was investigated and MT1-MMP-mediated intracellular uptake of pPAuNC was subsequently confirmed by a co-localization analysis using confocal microscopy and molecular competition test. Furthermore, we confirmed a change in the intracellular lipophilic network after an endocytosis event of pPAuNC. The identical lipophilic network change did not occur with the endocytosis of bare PAuNC. By classification of the branched network between the lipophilic organelles at the nanoscale, the image-based analysis of cell organelle networking allowed the evaluation of nanoparticle internalization and impaired cellular components after intracellular accumulation at a single-cell level. Our analyses suggest a methodology to achieve a better understanding of the mechanism by which nanoparticles enter cells.

## Introduction

Understanding of the interaction between nanoparticles and cancer cells is a kernel challenge to the development of nanomedicines for effective cancer diagnosis and treatment [1, 2]. In particular, it is important to identify intracellular changes caused by the endocytosis of nanoparticles through the cellular membrane ligand [3, 4]. Endocytosis does not simply indicate that nanoparticles have been introduced into the cytosol but also entails dynamic remodeling of cell membrane structures or internal vesicles [5–7]. Previous most studies have focused on whether the treated nanoparticles are located in the cytosol. However, research analyzing the intracellular lipophilic network transformation that accompanies endocytosis is still vague [8–10]. To explain the transformation of numerous lipophilic structures in cells, a systematic approach through imaging and morphological analysis of the intracellular organelle membrane structure is required. To remedy inadequate investigation of intracellular action in the development of nanoparticle-based therapeutics, a stable molecular probe that can provide visible information and prognostic diagnosis of cancer cells is required.

The preparation of pH-stable and biomarker-targetable nanoprobe is particularly important in the detection of cancer cells, which have the superior proliferative ability at alkaline intracellular pH and adapt to extracellular acidosis [12]. Gold-derived nanoplatforms have been extensively studied as useful tools for bio-sensing, cellular labeling, and molecular imaging [11]. Especially, the Au nanocluster (AuNC) of dimension less than 3 nm, has been reported to serve as a fluorescent molecular probe with advantages of colloidal stability, water solubility, and biocompatibility [13, 14]. However, AuNCs cause toxicity by accumulating a large amount in cells or tissues such as the liver and spleen [15, 16]. The cytotoxic effect can be mitigated by coating the metal surface using a biocompatible ligand as a reducing agent [17–19]. Optical characterization of AuNCs protected by albumin generally shows emission in the range of 600–800 nm, suitable for applying sensitive molecular imaging agents [20]. Moreover, albumin can be functionalized by attaching specific peptides that target and bind biomarkers of interest to surface functional groups. These can serve as important drug delivery agents as a practical example of nanomedicine clinicalization, such as Nab-PTX [21]. For the advanced diagnosis of cancer, AuNCs can be functionalized with specific peptides that target and bind to biomarkers of interest.

**Scheme 1:**
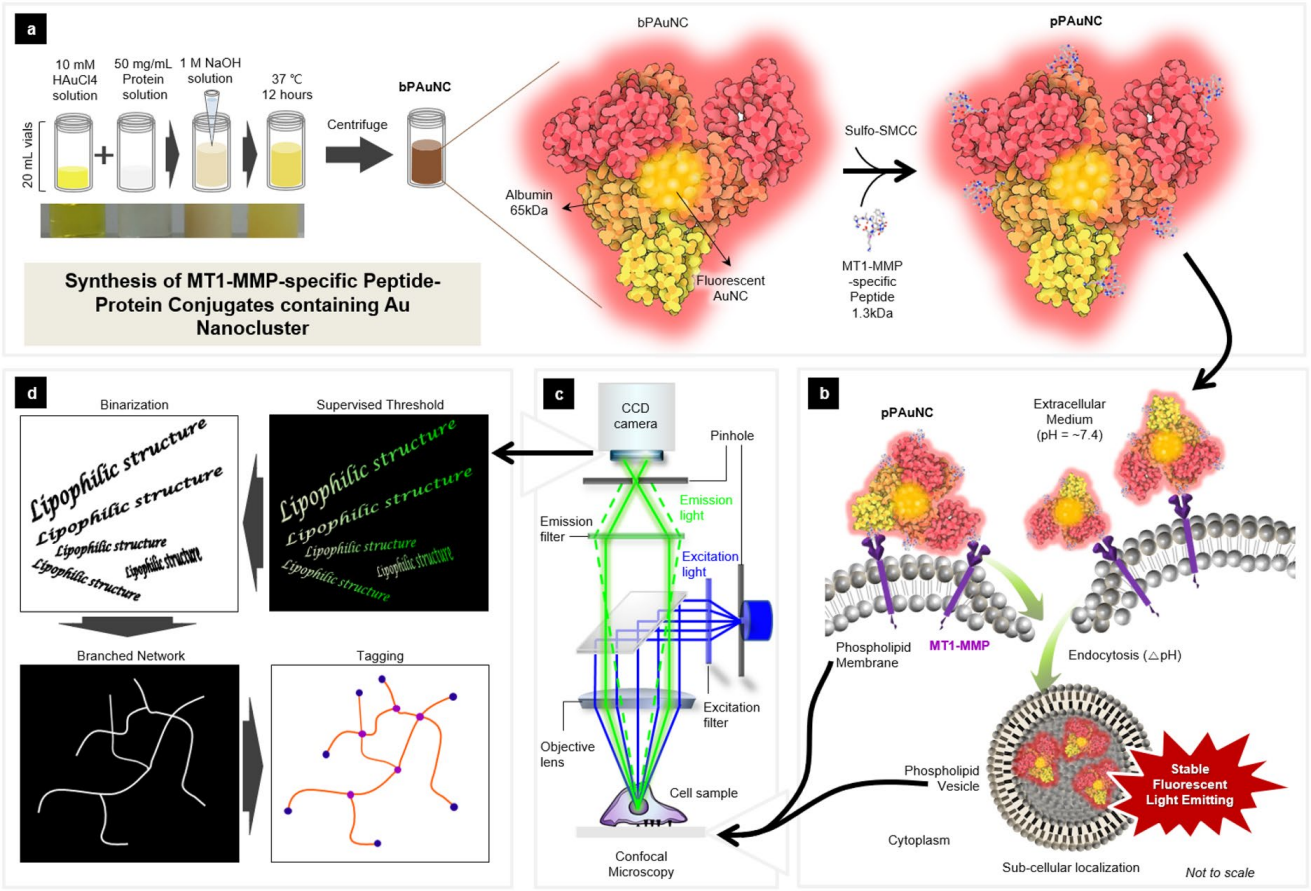
**a** Schematic illustration for the synthesis of MT1-MMP-specific peptide-conjugated protein-Au nanoclusters (pPAuNC). **b** The proposed endocytosis cascade for the sub-cellular localization of pPAuNC. **c** Single-cell microscopic analysis for the intracellular lipophilic network transformation after MT1-MMP-mediated endocytosis of pPAuNC

MT1-MMP, a membrane-type I matrix metalloproteinase, is known as a critical indicator of metastatic cancer and it degrades the surrounding extracellular matrix by expressing at the cancerous cell surface [22]. The trafficking of MT1-MMP-mediated endocytosis is crucial for an appropriate understanding of the cellular function dependent on metalloproteinase [23–25]. In this study, we thus developed pH-stable photoluminescent molecular probes based on AuNCs modified with MT1-MMP-specific cleavable peptide (pPAuNC) using albumin as a template to evaluate the cellular influx process through MT1-MMP (Scheme 1a). The colloidal photo-stability of pPAuNC was characterized by dynamic light scattering (DLS), atomic force microscopy (AFM), UV–visible spectroscopy, and fluorescence spectroscopy. To examine cytotoxicity, we conducted a crystal violet assay and cell morphological analysis. The biomarker-specific intracellular uptake and targeted imaging potential of pPAuNC were evaluated by co-localization analysis and competitive binding inhibition assay via confocal microscopic images using high MT1-MMP-expressing MDA-MB-231 and low MT1-MMP-expressing MCF7 cells (Scheme 1b, c). In addition, branched network analysis was performed to evaluate the lipophilic membrane structural alteration of intracellular organelles following the influx of bPAuNC or pPAuNC by MT1-MMP expression at the single-cell level (Scheme 1d). Here, we present “peptide-modified protein-based AuNCs (pPAuNCs)” as promising fluorogenic molecular probes for biomarker-specific fluorescence imaging. A distinct evaluation process through image-based investigation has been proposed to explain the behavior of nanoparticles after endocytosis. Furthermore, lipophilic network analysis of nanoparticle-treated cells will provide a new basis for determining how nanoparticles entering a cell may differ depending on the pathway of influx through receptor-mediated uptake or passive diffusion across the membrane.

## Experimental

### Reagents and materials

Gold(III) chloride trihydrate (HAuCl_4_·3H_2_O, 99.9% #520918) was purchased from Sigma Aldrich (St. Louis, MO, USA). Sodium hydroxide (NaOH, #0583) and Bovine serum albumin (BSA) was purchased from AMRESCO (Solon, OH, USA). Sulfosuccinimidyl 4-[N-maleimidomethyl]cyclohexane-1-carboxylate (Sulfo-SMCC, #22122) was purchased from Thermo Scientific (Rockford, lL, USA). Thiol-modified MT1-MMP-specific cleavable peptide (sequence: GPLPLRSWGLK) was purchased from Peptron (Republic of Korea).

### Synthesis of peptide-protein nanoconjugates

5 mL aqueous solution of BSA (250 mg mL^−1^) was added to 5 mL aqueous solution of HAuCl_4_ (10 mM) under magnetic stirring in the 30 mL vial at 37 ℃ for 2 min. Add 0.5 mL NaOH solution (20 M) was dropped into vial under stirring 37 ℃ for 12 h in a water bath incubator. After the reaction was completed, the mixture solution was filtered using 3 kDa cut-off Amicon Ultra-15 Centrifugal Filter Units (#UFC900324; Merck Millipore, Darmstadt, Germany) for 30 min at 4,000 g to remove impurities. Until the pH range was reached at 7.4, the centrifugation procedure was repeated with PBS (#LB001-02; Welgene, Republic of Korea) added at 30 min intervals. The filtrated solution was re-dispersed in PBS (pH 7.4). The thiol-modified MT1-MMP-specific cleavable peptides (0.8 µM) were reacted with sulfo-SMCC linker (0.8 µM). The BSA-stabilized gold nanocluster (bPAuNC, 0.1 µM) was subsequently added to the modified peptide solution. After 30 min, the final solution was filtrated and re-dispersed in PBS (pH 7.4) as a photoluminescent Au-based fusion protein probe (pPAuNC).

### Characterization

The absorption and fluorescence spectra were measured using a UV–Vis spectrophotometer (Lamda 25; Perkin Elmer, Waltham, MA, USA) and a fluorescence spectrometer (FP-6500; JASCO, Easton, MD, USA), respectively. The photoluminescence image was obtained after irradiation with UV lamp (wavelength; 350 nm). The size distribution of bPAuNC was analyzed by atomic force microscope (AFM, BioScope Resolve; Bruker, Billerica, MA, USA) and dynamic scattering spectrometer (DLS, ELSZ-2000, Otsuka; Japan).

### pH-dependent colloidal stability

Few drops of HCl and NaOH solution (1M stock solution) was adjusted to bPAuNC solution. The pH-dependent colloidal stability of bPAuNC was examined in the wide pH range from 4.04 to 12.06 and determined by visual inspection of a coagulation and fluorescence intensity.

### Cell culture

MDA-MB-231 (high MT1-MMP expression) and MCF7 (low MT1-MMP expression) cells, human breast cancer cell lines, were obtained from the Korean Cell Line Bank (Korean Cell Line Research Foundation, Seoul, Korea) and cultured in RPMI1640 (Gibco, Carlsbad, CA, USA) supplemented with 10% FBS (Gibco) at 37 ℃ and 5% CO_2_.

### Cytotoxicity assay

MDA-MB-231 and MCF7 cells were seeded into 24-well plates (5 × 10^4^ cells well^−1^). After 24 h, the cells reached 60–70% confluence, different concentration of bPAuNC and pPAuNC were treated to cells by serial dilution. The cellular viability was evaluated using a colorimetric assay based on the colony formation assay by crystal violet (CV, #V5265; Sigma-Aldrich) staining assay following the manufacturer’s instructions. The images were quantified using ImageJ software.

### In vitro uptake and targeting ability

MDA-MB-231 and MCF7 cells were seeded on 6-well plates (1 × 10^5^ cells well^−1^) and treated bPAuNC and pPAuNC after 24 h. For 4 h, the images were taken by a live cell imaging system (DMI6000B, Leica Microsystems). The protease targeting ability of bPAuNC and pPAuNC was assessed with immunofluorescence staining and confocal microscopy. The bPAuNC and pPAuNC-treated cells were fixed with 4% PFA (Biosesang, Republic of Korea) and permeabilized with 0.05% Triton X-100 (#T9285; Sigma-Aldrich). The cells were co-stained with the MT1-MMP antibody (#ab51074; abcam) and Hoechst33342 (#H3570; ThermoFisher Scientific). The confocal microscopic images were captured by LSM700 (Carl Zeiss, Jena, Germany) and analyzed by the ZEN software. For visualizing and measuring the co-localization of MT1-MMP and AuNC in cells, the correlation value was evaluated by a Fiji program plugin. The correlation measurements of intracellular co-localization were quantified intensity profiles across the lines at a single cellular level.

### Competitive binding assay

To inhibit the binding and endocytic uptake through albumin by increasing extracellular concentrations of unlabeled BSA, the MDA-MB-231 cells were incubated with 2% BSA-contained solution for 4 h before the bPAuNC and pPAuNC treatment. For tracing of competitive binding interaction with intracellular components, intracellular lipophilic structures were labeled by CLLD lipophilic tracer dye (#D7757, ThermoFisher Scientific). The co-localization of CLLD and bPAuNC or pPAuNC were determined by a Fiji program.

### Intracellular branched network analysis (IBNA)

The interaction with endocytic components of bPAuNC and pPAuNC after internalization was identified by the intracellular branched network analysis (IBNA). The single-cell image was binarized and skeletonized for visualizing the local morphological information with lipophilic structure. For categorizing branched patterns of skeletonized structure, the network junctions were tagged in a different color depending on the number of neighbors using a Fiji program.

## Results and discussion

### Photo-stability of protein-based Au nanocluster (PAuNC)

**Fig. 1:**
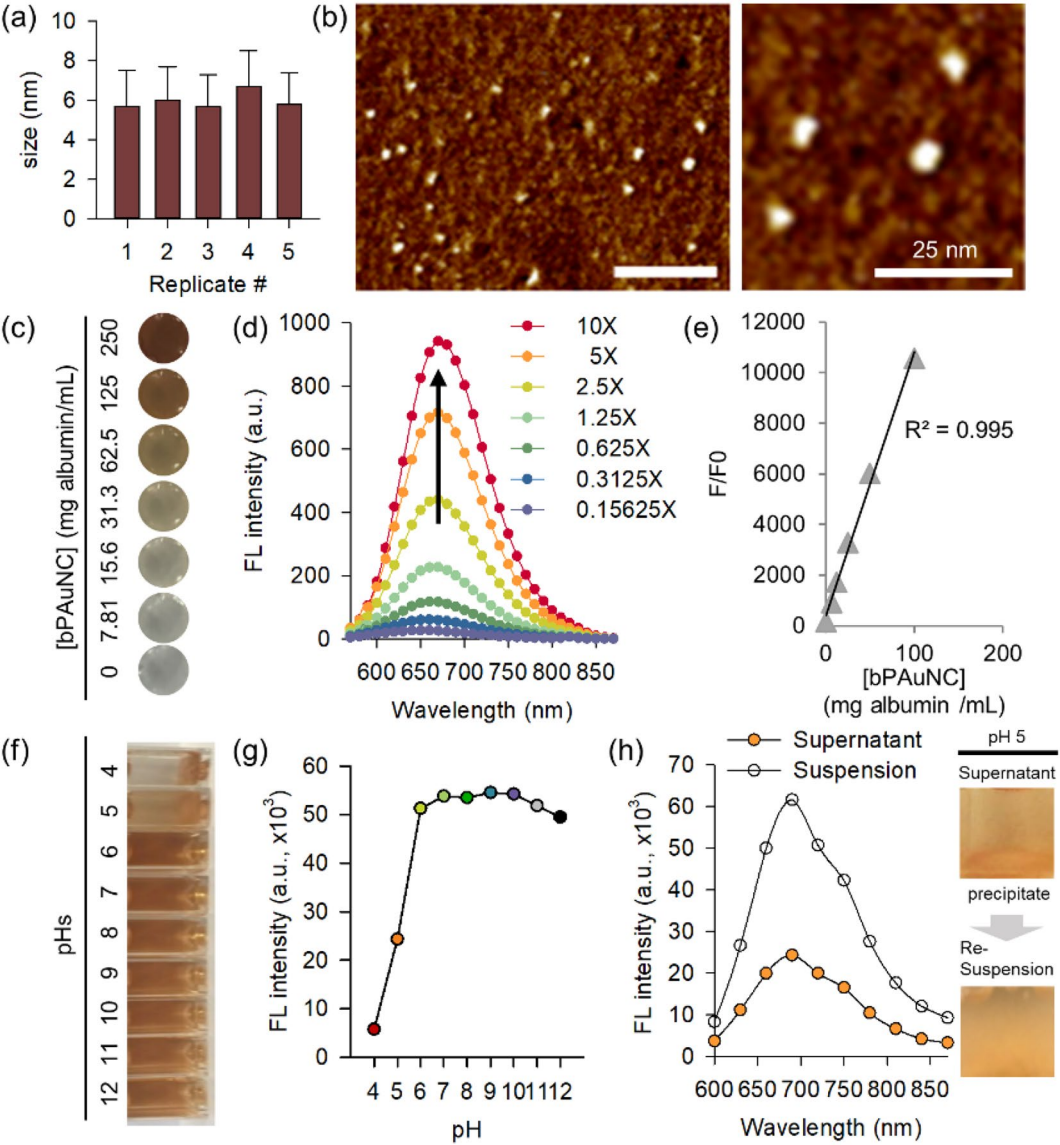
Characterization and pH-dependent colloidal stability of bPAuNC. **a** Colloidal size of bPAuNC repeatedly measured by DLS. **b** AFM images of bPAuNC (left) and the magnified microscopic image (right). Scale bar is 50 nm. **c** Photographs of bPAuNC containing various concentrations (3.91, 7.81, 15.63, 31.25, 62.5, 125, 250 mg mL^−1^) of albumin. **d** Fluorescence emission spectra of bPAuNC depending on albumin concentration (1X = 125 mg albumin mL^−1^). **e** Linear relationship between the normalized fluorescence intensity of bPAuNC (F/F0) and the concentration of albumin. **f** Photographs of bPAuNC solution (125 mg albumin mL^−1^) depicting colloidal stability agglomeration state of bPAuNC under different pH values by addition of HCl (0.1 M) or NaOH (0.1 M) to the solution. The pH ranges from 4.041 to 12.064. **g** Fluorescence decay of the bPAuNC affected by the pH value of the reaction solution. **h** The separated supernatant or re-suspended bPAuNC solution according to its change in solubility at pH 5 as analyzed by fluorescence spectra. Inset of **h**: Photographs of the separated supernatant (left) and resuspended bPAuNC solution (right) at pH 5

Fluorescence protein-Au nanoclusters (PAuNC) were prepared using albumin as a stabilizer and reducing according to the protocol developed by previous research [26, 27]. PAuNC stabilized by clustering inside the albumin molecules was evaluated by dynamic light scattering (DLS) and atomic force microscopy (AFM). The hydrodynamic diameter of bare PAuNC (bPAuNC) was repeatedly measured and the indicated average colloidal size was smaller than 6 nm (Fig. 1a). As shown in Fig. 1b, the nanometer-scale bPAuNC was also confirmed by AFM-mediated topology scanning after the drying. The AFM images for bPAuNC represented uniformly dispersed spherical shapes without critical aggregation. In addition, colloidal stability was maintained even when bPAuNC was diluted with phosphate-buffered saline (PBS) (Fig. 1c). The photoluminescence of the synthesized bPAuNC was exhibited due to due to different distinct electronic transitions from their HOMO–LUMO band gap [28]. The fluorescence spectra of diluted bPAuNC solution in Fig. 1d were increased by concentration but exhibited no spectral shift. The maximum emission wavelength is at 670 nm, and the relative fluorescence enhancement plotted as F/F0 was considered to be linearly correlated to bPAuNC concentration (R^2^ = 0.995, Fig. 1e). We further demonstrated that bPAuNC maintains fluorescence stability under pH-variation without fluorescence-quenching, thus confirming its applicability in molecular imaging. Specifically, lowering the pH from 7.0 to below the isoelectric point gradually unfolds the BSA structure, increases the molecular size, and enhances the relative content of the β-sheet. This unfolded structure promotes the formation of insoluble aggregates in the solution and increases the instability of BSA [29]. The pH was adjusted to the desired value (pH 4 to 12) by adding 1 M HCl or 1M NaOH to the prepared stock solution. The photographs of the bPAuNC solution at each pH value demonstrate the colloidal stability and show its collapse at pH 5 due to particle aggregation (Fig. 1f). The dispersion instability of bPAuNC arose at the isoelectric point (IEP) of protein. The IEP for albumin was found to be at pH 5.07 and protein aggregation at the gas–liquid interface is known to form insoluble matter in the pH range lower than 5 [29]. We confirmed that albumin aggregation with pH changes also affected the optical properties of bPAuNC. The fluorescence intensity maximum at 670 nm did not show a significant difference from pH 6 to 12 but decreased rapidly from pH 5 (Fig. 1g). However, the fluorescence spectra of the supernatant separated by particle aggregation and the resuspended solution at pH 5 still showed maximum emission at 670 nm (Fig. 1h). The interactions between nanoparticles and targeted cells were affected by numerous biological processes during the entire endocytosis process [30, 31]. The participating organelles at each stage in the endocytosis pathway are more acidic, unlike typical cytosolic pH (~ 7.2). The pH value decreases to ~ 6.3 in the early endosome, ~ 5.5 in the late endosome, and 4.5 or less in the lysosome [32, 33]. Therefore, these results indicate that bPAuNC, which maintains fluorescence at pH 5, is suitable for tracking nanoparticle uptake in the cellular environment due to the maintaining of high quantum yield. These results showed the high stability and bioavailability of bPAuNC for fluorescence imaging probes.

### Biocompatibility evaluation of MT1-MMP-specific peptide-conjugated PAuNC (pPAuNC)

**Fig. 2:**
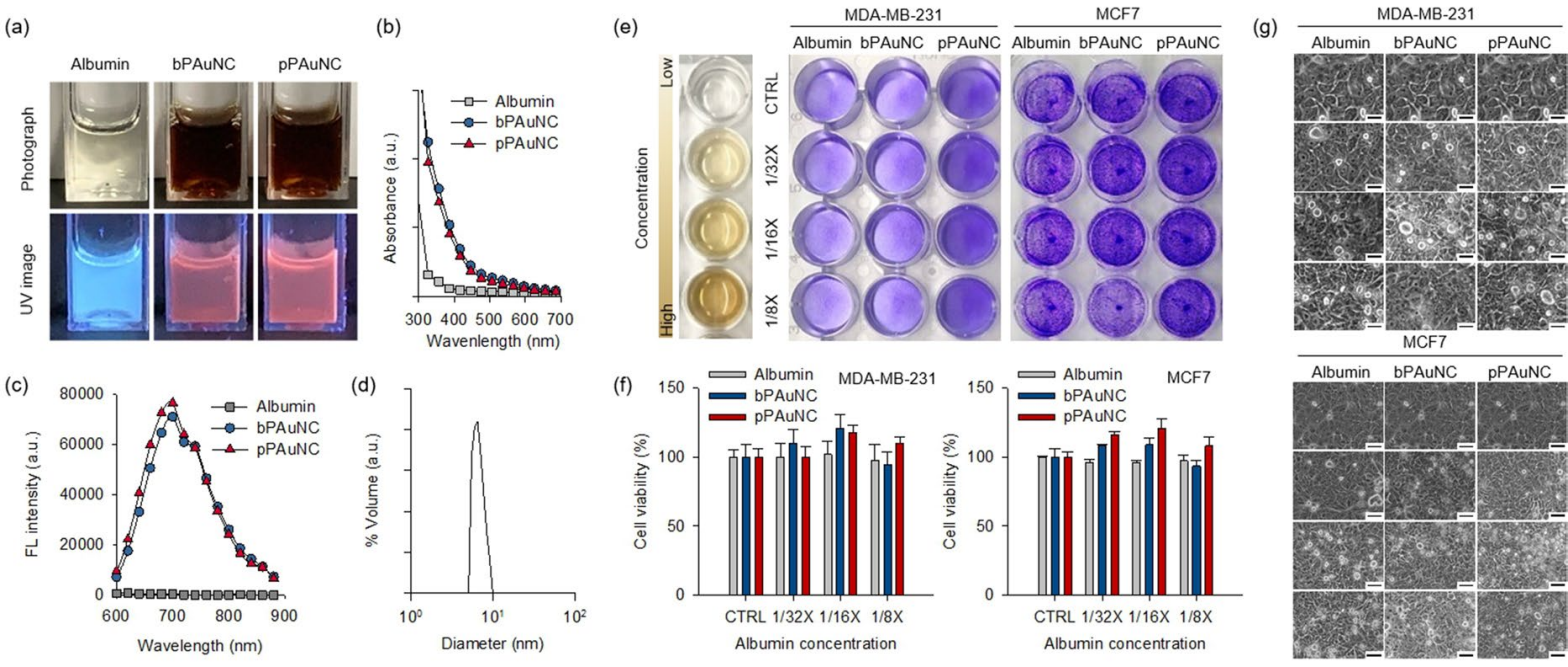
In vitro biocompatibility of bPAuNC and pPAuNC. **a** Photographs of pre (upper) and post (lower) irradiation with UV (350 nm) of albumin, bPAuNC, and pPAuNC. **b** Absorption and **c** fluorescence spectra of albumin, bPAuNC, and pPAuNC (125 mg albumin mL^−1^). **b** Size distribution of pPAuNC by dynamic laser scattering. **e** MDA-MB-231 (high MT1-MMP expression) and MCF7 (low MT1-MMP expression) breast cancer cell lines incubated for 24 h with albumin, bPAuNC, and pPAuNC at various concentrations. Crystal violet proliferation assay to assess the cytotoxic effect of the albumin, bPAuNC, and pPAuNC on cell growth inhibition. **f** Cell viability and **g** microscopic phase images of cells treated with albumin, bPAuNC, or pPAuNC for 24 h incubation time. Scale bars are 50 µm

In the field of nanomedicine applied to cancer treatment, efficient detection of biomarkers and nanotoxicity are important issues. The metastasis of cancer cells is a crucial challenge in cancer research. We designed a photoluminescent imaging sensor for imaging cancer cells with metastatic properties and evaluated its suitability for bio applications. Peptide-protein conjugated AuNCs (pPAuNC) were synthesized using specific peptides targeting the protease, MT-MMP, which is necessary for cancer cells to dissolve the surrounding substrate for migration and invasion. Albumin contains 35 thiol groups, 99 carboxylic groups, and 120 amino groups which are linking sites that offer potential for conjugation. One end of the MT1-MMP specific cleavable peptides (GPLPLRSWGLK) of our group was modified into a thiol group and then reacted with amine groups on the albumin surface of PAuNC using sulfo-SMCC linker [34]. To investigate the fluorescence potential of the pPAuNC, UV light irradiation and fluorescence spectroscopy were carried out. Figure 2a exhibits the corresponding photographs of the albumin, bPAuNC, and pPAuNC taken under white light (upper) and UV light (λ = 350 nm) irradiation conditions. In the case of a transparent albumin solution, UV light was transmitted to give a blue color, but red fluorescence was emitted from the brown-colored bPAuNC and pPAuNC (Fig. 2b). pPAuNC displayed a characteristic emission peak at 670 nm, analogous to that of bPAuNC (Fig. 2c). Moreover, there is no critical change for the average size of pPAuNC (5.96 ± 1.06 nm) compared to non-functionalized bPAuNC (Fig. 2d). The photoluminescent characteristics of pPAuNC were maintained even after peptide conjugation with bPAuNC. The cytotoxicity of albumin, bPAuNC, and pPAuNC were evaluated using crystal violet staining and morphological change of the cancer cells. MDA-MB-231 cells with high MT1-MMP expression and MCF7 cells with low MT1-MMP expression were used as model cell lines to assess growth inhibition according to biomarker selectivity. Considering that the concentration of the solution buffer (PBS) contained in the growth medium does not affect the cell survival, serially diluted albumin, bPAuNC, and pPAuNC were treated at 24 h after cell seeding. As compared to albumin treatment, crystal violet staining revealed increased cell proliferation with treatment by bPAuNC or pPAuNC in both cell lines (Fig. 2e, f). In particular, MDA-MB-231 cells had higher cell viability after pPAuNC treatment than any other conditions. This increased viability was also captured by photographs of the cell morphology (Fig. 2g). We speculate that albumin used in the gold nanoclusters entered the cells and supported cell growth as a desirable factor. Albumin is known to offer broad support in mammalian cell growth as an important carrier of serum-derived substances including lipids, amino acids, hormones, peptides, metals, and other low molecular weight molecules [35]. Therefore, these results indicate the chemical and structural benefits resulting from the systemic modification of albumin-based gold nanoclusters and point toward the potential of bio-applications of pPAuNC.

### Correlation of MT1-MMP expression levels with intracellular uptake of pPAuNC

**Fig. 3:**
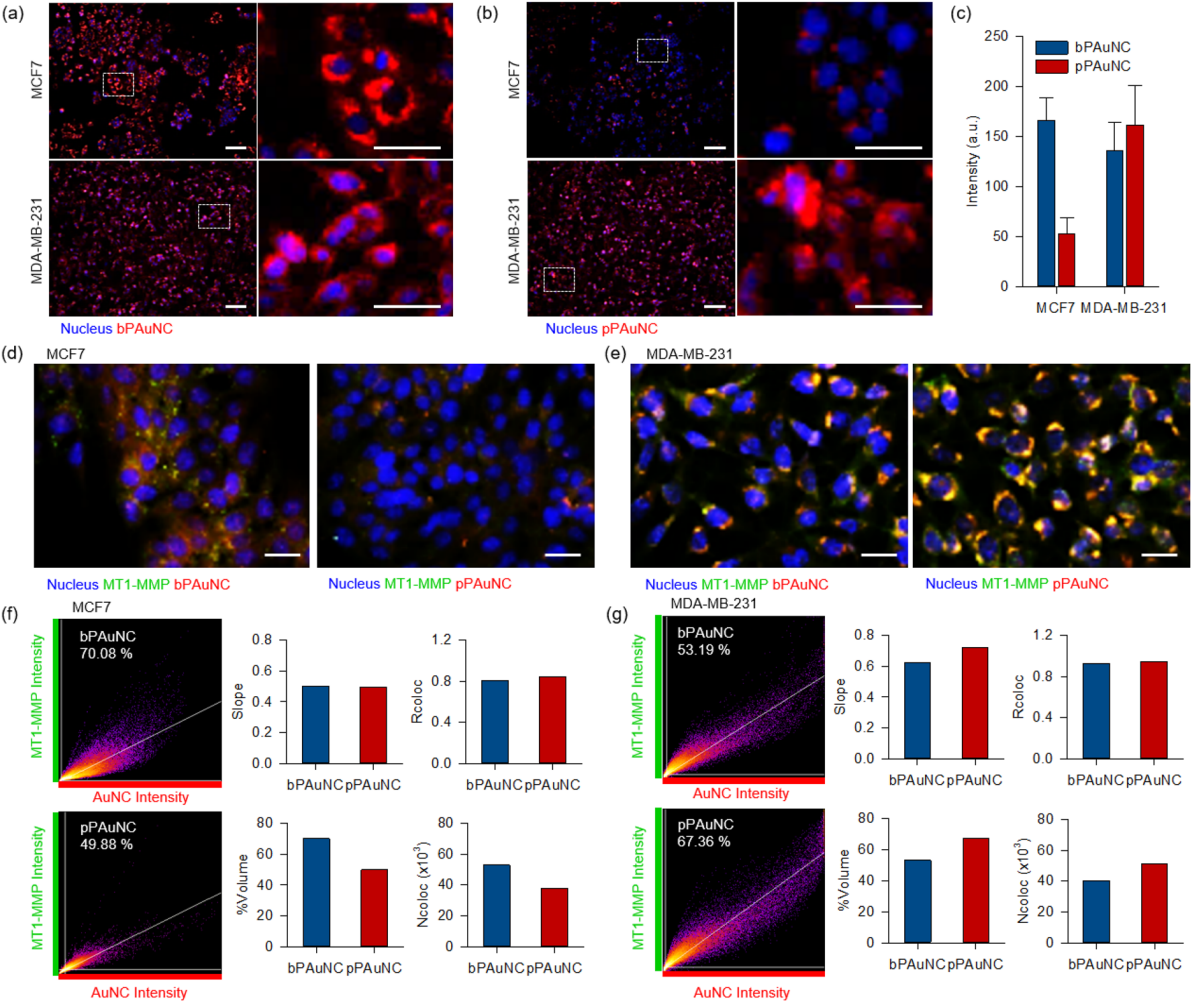
Comparison analysis for the intracellular uptake and targeting potential of bPAuNC and pPAuNC. Fluorescence microscopic images of MCF7 (low MT1-MMP expression) and MDA-MB-231 (high MT1-MMP expression) breast cancer cell line incubated for 10 h with **a** bPAuNC and **b** pPAuNC. Magnified region depicted in the white dashed-line box and scale bars are 100 µm. **c** Quantification graph for the fluorescence intensity measured from confocal microscopic images for **d** MCF7 and **e** MDA-MB-231 cells treated with bPAuNC or pPAuNC (red); MT1-MMP (green) and Hoechst33342 (blue). Scale bars are 50 µm. Co-localization analysis between MT1-MMP (green) and bPAuNC or pPAuNC (red) in MCF7 **f** and MDA-MB-231 **g** were depicted as scatterplots. Pearson’s coefficient and correlation values were generated using ImageJ; Slope, Rcoloc, %Volume, and Ncoloc

For further validation of stable synthesis and target selectivity of pPAuNC, a live cell imaging system was utilized. To investigate whether MT1-MMP expression levels correlate with endocytosis of pPAuNC, the MCF7 (MT1-MMPlow) and MDA-MB-231 (MT1-MMPhigh) cells were treated with bPAuNC and pPAuNC. Nontoxic concentrations of bPAuNC and pPAuNC (125 mg albumin mL-1) were chosen by examination of Fig. 2d. Uptake and intracellular trafficking of the fluorescence of bPAuNC and pPAuNC were detected in real-time. As a result, we confirmed that the fluorescence signal of bPAuNC and pPAuNC was adequately persistent inside the cells 10 h after the nanoparticles were treated. Figure 3a, b shows the internalization of bPAuNC at the cytosol of both cells. In contrast, the functionalized pPAuNC for MT1-MMP-specific targeting was more internalized into MDA-MB-231 cells and resulted in a three-fold increase of fluorescence intensity compared to MCF7 cells (Fig. 3c). To confirm MT1-MMP-specific endocytosis pathway involved in pPAuNC uptake, we stained MCF7 and MDA-MB-231 cells with the MT1-MMP antibody and analyzed co-localization values of fluorescent bPAuNC or pPAuNC and the MT1-MMP channel. According to fluorescence microscope images, the signals released from internalized bPAuNC or pPAuNC were co-localized with green-stained MT1-MMP. Merged fluorescence signals appeared as orange and yellow. In the case of MCF7 cells, the fluorescence signal of bPAuNC introduced into the cell is strong, but there are many regions of red or green fluorescence indicating that bPAuNC does not coexist with MT1-MMP (Fig. 3d). The co-localized signals of pPAuNC and MT1-MMP were also decreased. However, significant uptake of pPAuNC and increase in the co-localized signal intensity was observed in MDA-MB-231 cells, which indicates MT1-MMP-mediated internalization (Fig. 3e). Co-localization quantification was performed by the scatter plot divided into four quadrants with automatic thresholding by the imaging processing. The relative assessment of co-localization between MT1-MMP and bPAuNC or pPAuNC was revealed by four values from the scatter graph; the slope of the linear regression line (slope), Pearson’s correlation coefficient for pixels where both fluorescence channel intensities peak above the threshold of the scatter plot (Rcoloc), the number of pixels which have both channel intensities above the threshold of the scatter plot (Ncoloc), and the percentage of the Ncoloc over the total number of pixels in the image (%Volume). Pearson's correlation coefficient is a popular method of quantifying correlation in many fields of research ranging from psychology to economics. A value close to one for Pearson’s correlation coefficient for fluorescent images indicates reliable co-localization [36–38]. As expected, %Volume and Ncoloc values of the co-localized MT1-MMP and pPAuNC expressed the co-localized voxels were decreased in MCF7 cell images and increased in MDA-MB-231 cell images compared to bPAuNC co-localized signal. Both slope and Rcoloc displayed similar values in interactions of MT1-MMP with bPAuNC or pPAuNC, but the pPAuNC and MT1-MMP co-localization regions were very limited on the scatter plot of MCF7 cells (Fig. 3f). However, in MDA-MB-231 cells, the increased slope and Rcoloc values with pPAuNC were noticeable in comparison to the co-localization regions with MT1-MMP and bPAuNC or pPAuNC in the scatter plot (Fig. 3g).

**Fig. 4:**
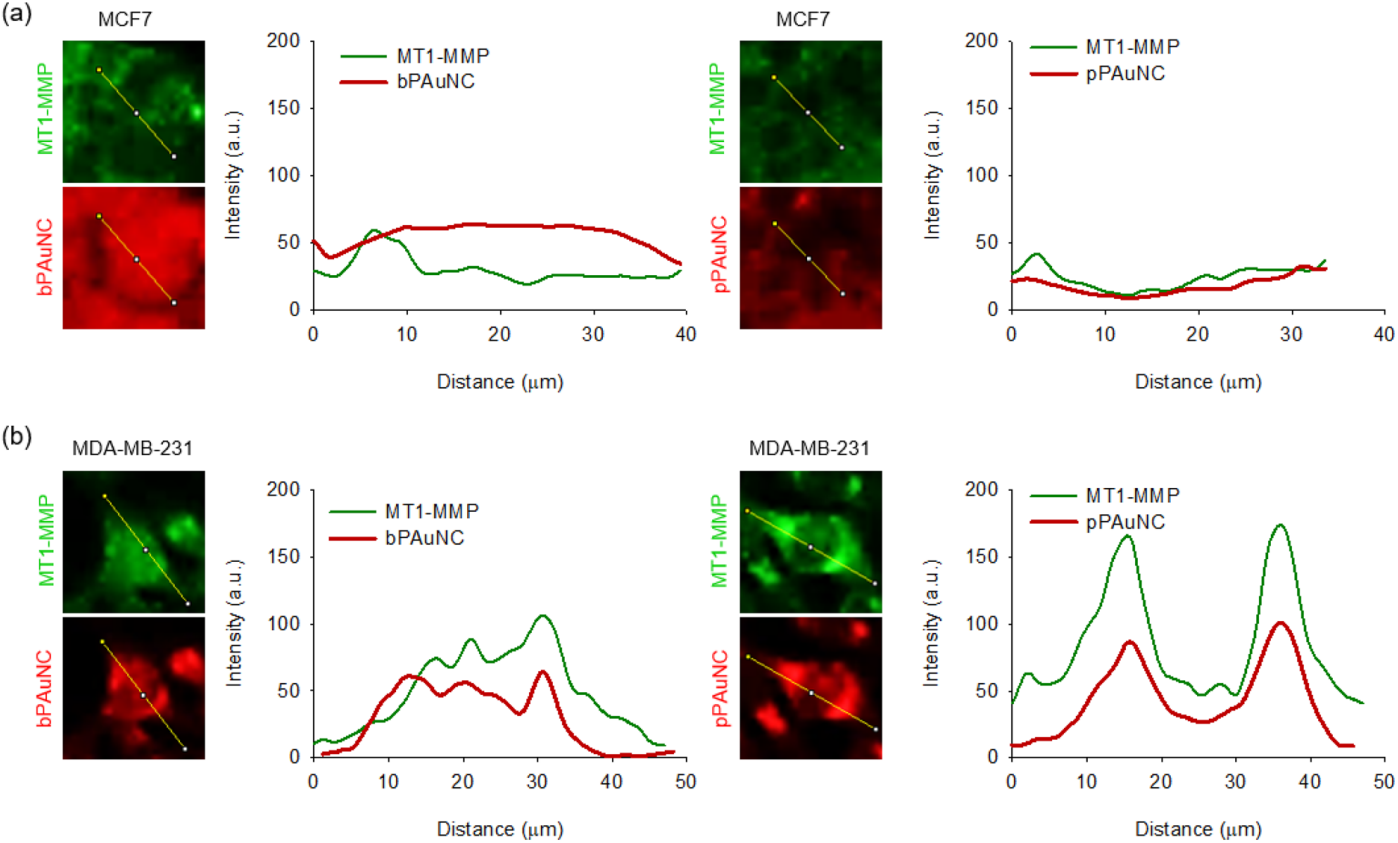
Co-localization analysis between MT1-MMP and bPAuNC or pPAuNC at a single-cell level. The fluorescence intensity profiles were quantified along a line in the image of **a** MCF7 and **b** MDA-MB-231 cells incubated with bPAuNC or pPAuNC (red) and co-stained with MT1-MMP antibody (green)

Most image-based cell-profiling workflows essentially assume that composite averages of single-cell measurements reflect the dominant biological mechanism influenced by external stimulation [39]. Interpretation and visualization of single-cell profiling data are very important because subpopulations of cells may exhibit different phenotypes under the same conditions. Thus, we confirmed that co-localization analysis in the total image area was consistent without distortion even when applied at the single-cell level. For each cell, the lines were drawn across the entire width of cells in green (MT1-MMP) and red (bPAuNC or pPAuNC) fluorescence images. The two-dimensional graph of the intensities of pixels along a line within the image was displayed and represented the distance along the line (x-axis) and the pixel intensity (y-axis). These data showed that the green signal strength in MCF7 is significantly lower than in MDA-MB-231 and that most of the green signals of MT1-MMP do not overlap with the red signals of bPAuNC or pPAuNC (Fig. 4a). In contrast, the line profile plots of MDA-MB-231 cells treated with pPAuNC indicated almost completely matched intensity distribution patterns of green and red channels through the yellow line in the magnified view of ROI (Fig. 4b). These results demonstrated and quantified the level of MT1-MMP-induced endocytosis of pPAuNC by statistically significant changes in the co-localization response. Additionally, image-based approaches for co-localization analysis are useful for identifying receptor-mediated internalization.

### Inhibition of the intracellular uptake of pPAuNC

**Fig. 5:**
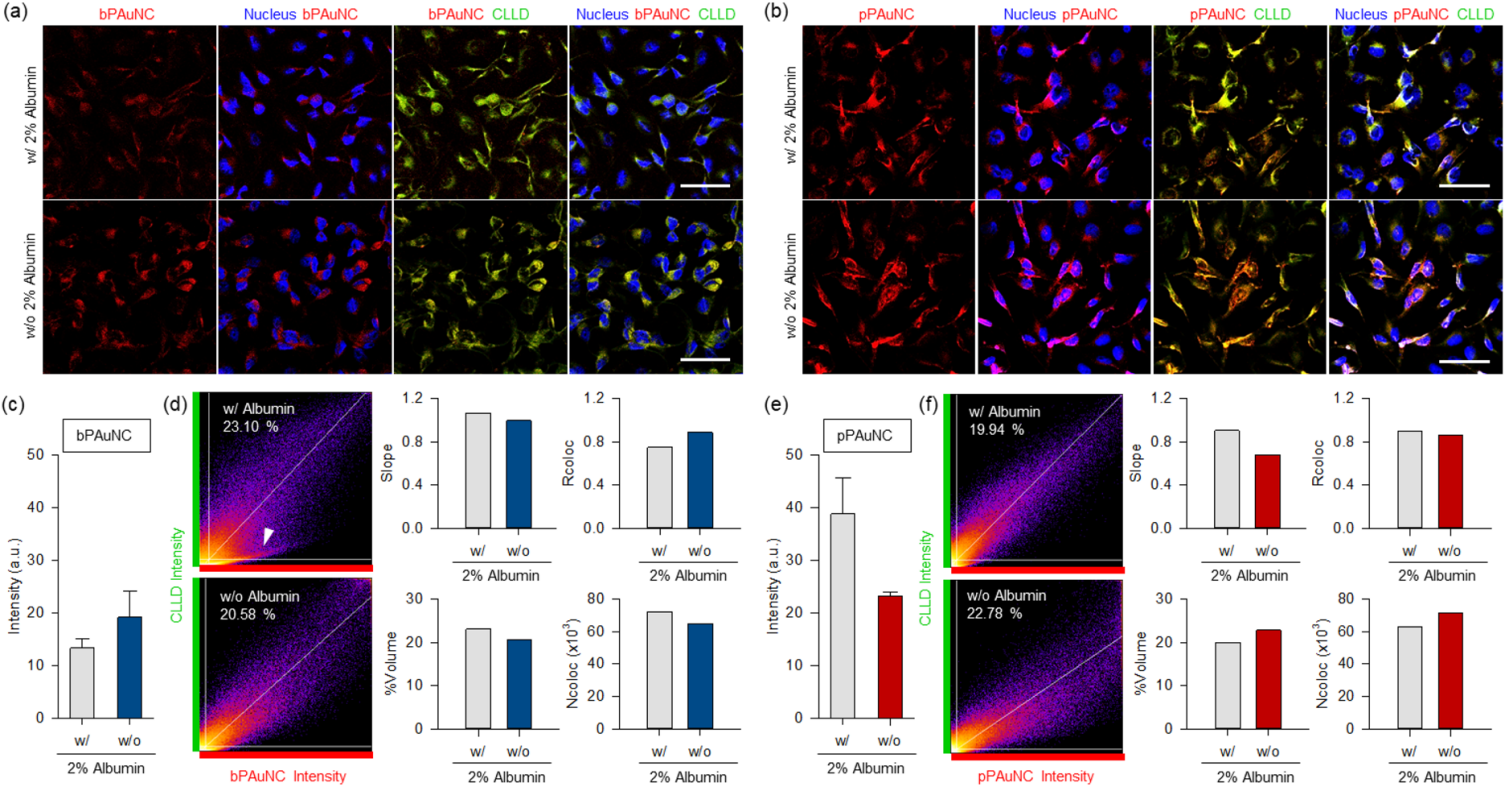
Competitive binding inhibition of bPAuNC and pPAuNC by increasing extracellular concentration of albumin in MDA-MB-231 cells. For binding inhibition, MDA-MB-231 cells were incubated with 2% albumin for 4 h; non-treated (w/o) and treated (w/). After albumin blocking, **a** bPAuNC and **b** pPAuNC were treated to MDA-MB-231 cells for 10 h; nucleus (blue), lipophilic membrane dye (CLLD, pseudocolor: green), bPAuNC or pPAuNC (red) and co-localization of CLLD and bPAuNC or pPAuNC (yellow). Scale bars are 50 μm. **c**, **e** Average fluorescence intensity of bPAuNC or pPAuNC in the cell cytoplasm. **d**, **f** Scatterplots and bar graphs through co-localization analysis of CLLD against bPAuNC or pPAuNC, respectively. Pearson’s coefficient and correlation values were generated using the ImageJ program; Slope, Rcoloc, %Volume, and Ncoloc

**Fig. 6:**
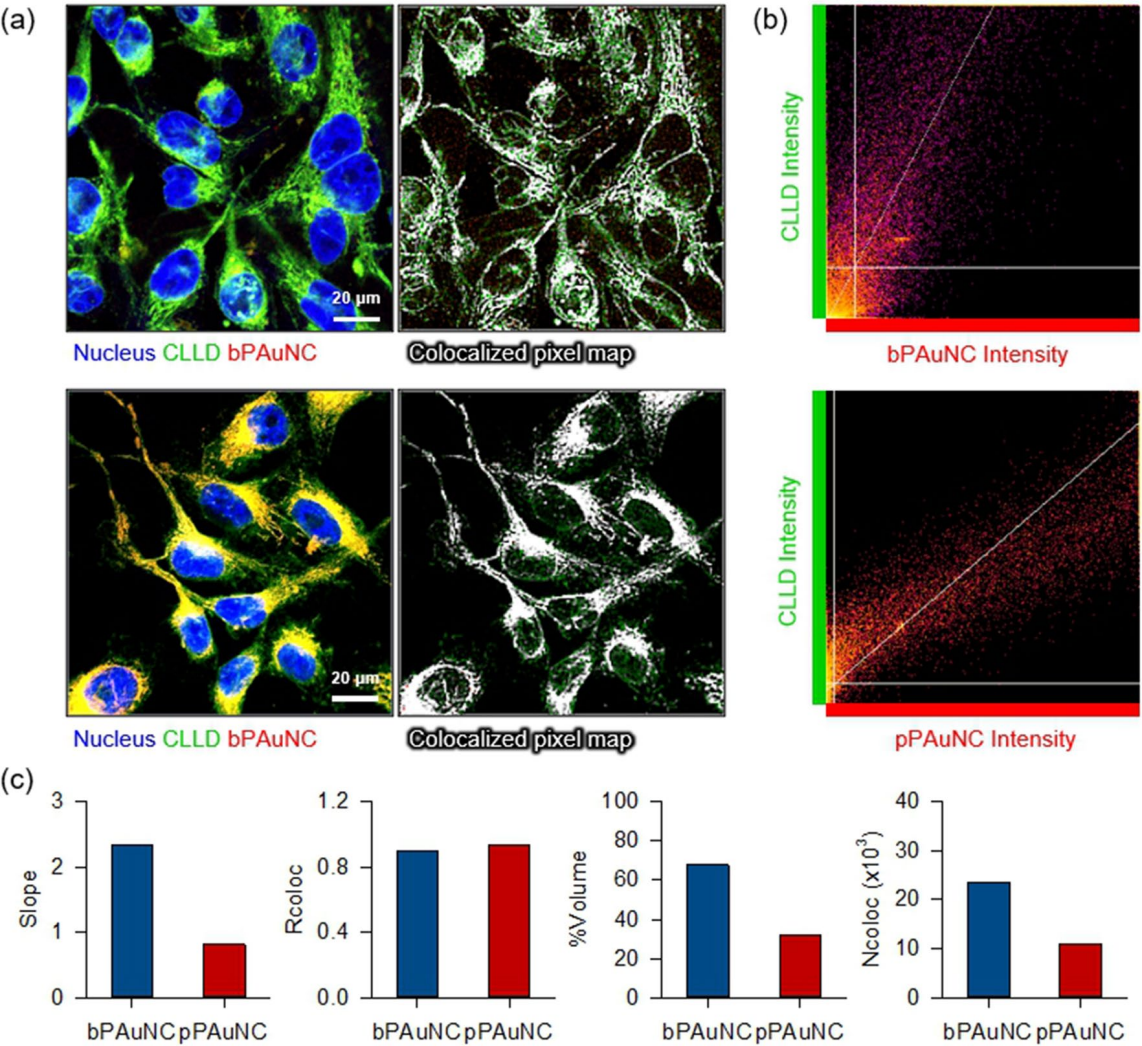
Co-localization analysis of lipophilic membrane-based organelle structure and bPAuNC or pPAuNC. **a** Confocal fluorescence images of MDA-MB-231 cells incubated for 10 h with bPAuNC or pPAuNC and co-stained with CLLD; nucleus (blue), lipophilic membrane dye (CLLD, pseudocolor: Green), bPAuNC or pPAuNC (red) and co-localization of CLLD and bPAuNC or pPAuNC (yellow). Co-localized pixel map shows the co-localization of CLLD and bPAuNC or pPAuNC as a white color. Scale bars are 20 μm. **b** The scatterplots were plotted against each color for co-localization analysis of bPAuNC or pPAuNC with CLLD and measured correlation coefficient in (c)

To determine the intracellular uptake mechanism of bPAuNC and pPAuNC, a competitive binding assay in the presence of free-albumin was conducted in MDA-MB-231 cells. MDA-MB-231 cells were incubated with 2% w/v albumin for 4 h followed by co-treatment with bPAuNC or pPAuNC for up to 10 h. The interaction with nanoclusters and subcellular compartments via endocytic vesicles is important to understand their intracellular trafficking pathways [3, 40]. Also, parameters like nanoparticle hydrophilicity and lipophilicity decide their uptake, accumulation, or release [41, 42]. To track the interaction with the intracellular components with lipophilic properties, 1,1'-dioctadecyl-3,3,3',3'-tetramethylindodicarbocyanine, 4-chlorobenzenesulfonate salt, a cellular lipophilic labeling dye (CLLD), was used to confirm intracellular migration of the particles after they attached to the cell membrane. As a lipophilic tracer, CLLD is highly fluorescent and quite photo-stable when incorporated into the membrane and does not affect cell proliferation [43]. As presented in Fig. 5a, intracellular uptake of bPAuNC decreased with increasing the extracellular concentration of albumin due to competition. Co-localization analysis with CLLD also confirmed that the red fluorescence of bPAuNC was scattered in cytosol except for the yellow region where fluorescence of CLLD and bPAuNC blended together, showing that bPAuNC depends on albumin-mediated recognition and endocytosis. In contrast, uptake of pPAuNC was not attributed to albumin-mediated endocytosis, as the inclusion of free albumin CLLD did not show an inhibitory effect on the amount of uptake (Fig. 5b). The fluorescence intensities of intracellular localized bPAuNC or pPAuNC were quantified in several random regions of each cell within the cytoplasm area gated by an imaging processing (Fig. 5c, e). The co-localization of CLLD and bPAuNC or pPAuNC in the cell was also evident. The scatter plots of red (pPAuNC) and green (CLLD) pixel intensities of the image exhibit the relative distribution of the points that existed around a straight line with or without extracellular albumin (Fig. 5f). Moreover, the co-localization of pPAuNC and CLLD demonstrated by the high slope and Rcoloc values appeared more extensively distributed in the case of the MT1-MMP-expressing MDA-MB-231 cells incubated with extracellular albumin for inhibiting albumin-mediated endocytosis. The %Volume and Ncoloc graphs show co-localized pPAuNC with CLLD in the without extracellular albumin was more spread in the scatter plot. In contrast, the decrease of co-localization of bPAuNC and CLLD with extracellular albumin in the upper image shown in Fig. 5a is represented by the distribution of points into two separate groups (white arrow in Fig. 5d). The saturated slope (> 1) and lower Rcoloc values were also considered to be caused by the weak correlation when compared with other conditions. The increase in the values of %Volume and Ncoloc showed that the populations of intracellular bPAuNC were not exactly overlapped with CLLD. To further explore the co-localization analysis with high magnification images, we analyzed the cells treated with bPAuNC or pPAuNC without albumin interference (Fig. 6a). Co-localized pixel maps displayed the overlapped fluorescence signal of CLLD and pPAuNC as white. Because the intensity of CLLD was relatively high compared to bPAuNC fluorescence, the slope value was saturated, whereas the pPAuNC and CLLD fluorescence intensity showed the slope and Rcoloc values approaching one (Fig. 6b, c). The %Volume and Ncoloc graphs revealed the dense distribution of co-localization between CLLD and pPAuNC around the correlation line. Therefore, the endocytosis process of pPAuNC in MDA-MB-231 cells may be considered the MT1-MMP-specific binding and mediated endocytosis of pPAuNC unaffected by extracellular albumin.

### Analysis of lipophilic network induced by the MT1-MMP-dependent endocytosis of pPAuNC at the single-cell level

As important as selecting the core materials and biomarkers of nanoparticles for cancer treatment is an understanding of how nanoparticles actually work within cells. Even if nanoparticles enter cells through an expected pathway, they interact with other organelles responsible for physiological properties inside the cell. Intracellular organelles have often been thought to act as structurally and functionally independent and autonomous [44]. However, recent studies have shown that organelles continue to communicate in order to maintain and regulate changes in cellular physiology and the environment caused by internal and external stimuli [45]. The endocytic process of engulfing nanoparticles is also related to the complex behavior of intracellular organelle networks. Cell organelles can be classified into non-membrane organelles such as ribosomes, centrosomes, and cytoskeletal structures, and membrane-bound organelles such as Golgi, ER, endosome, lysosome, mitochondria, and nucleus [46, 47].

**Fig. 7:**
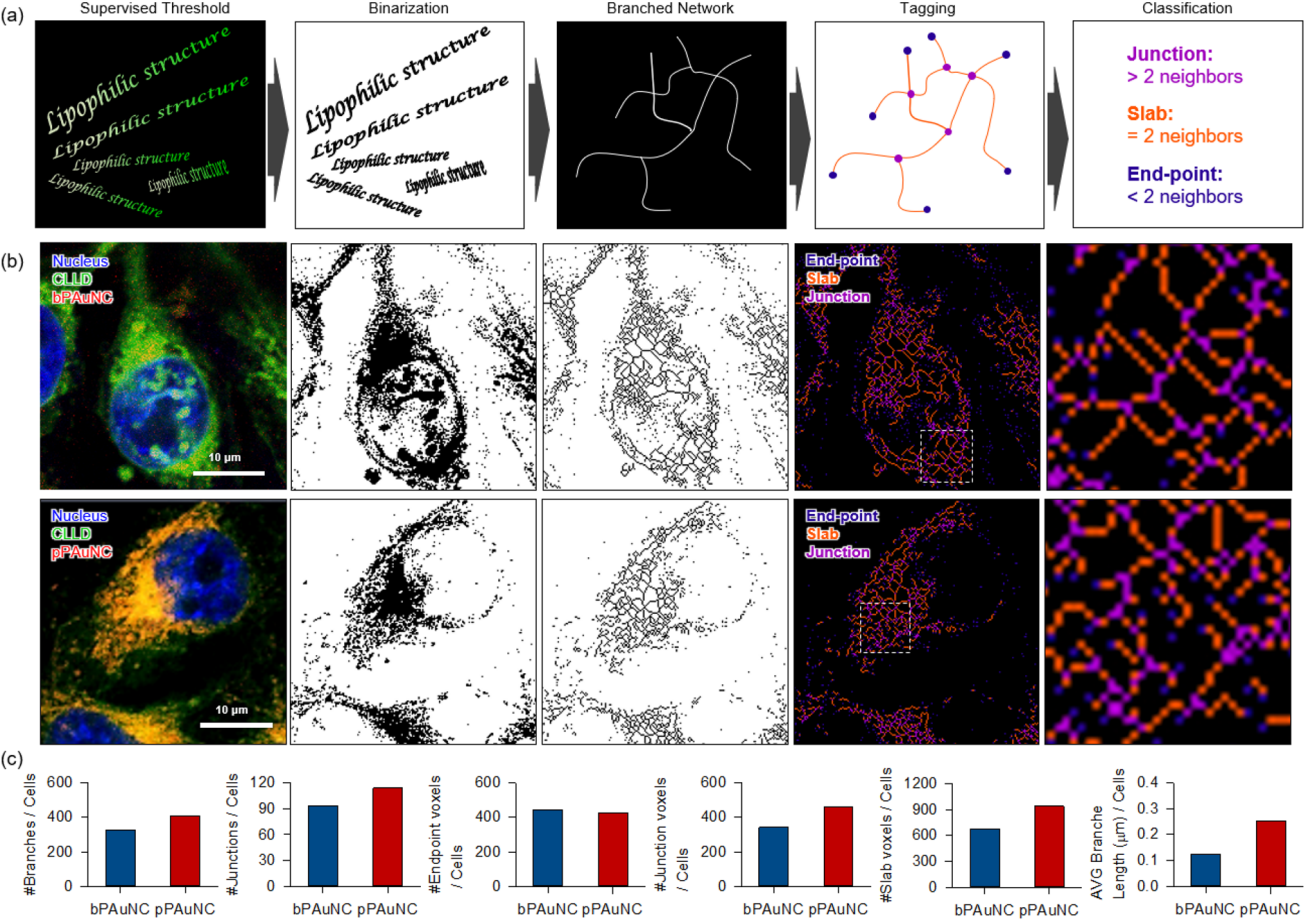
Image-based single-cell profiling of internalization of bPAuNC or pPAuNC by branched network analysis of intracellular organelles. **a** Schematic illustration of the intracellular organelle profiling process by intracellular branched network analysis (IBNA). **b** Classification of the branched network of lipophilic membrane-based organelles by three-colored tagging at the single-cell level. **c** The number of branches, actual junctions, and voxels of every type (end-point, slab, junction) and the average length of branches were quantified and displayed

Intracellular distribution of pPAuNC presented that MT1-MMP-expression dependent co-localization with CLLD. In most studies, the uptake and distribution routes of nanoparticles into/out from cells have been determined by endosome-lysosome markers [10, 48]. However, numerous intracellular organelles have extensive communication and organization for their physiological functions and to modulate the internal environment [49, 50]. Moreover, it is difficult to determine the exact time when the nanoparticles are trapped or for how long they stay in the endosome and lysosome, without a real-time screening assay. There have been issues with lysosome dysfunction or damage linked to morphological alterations like swelling and disruption due to the intracellular accumulation of nanoparticles [51–53]. Therefore, specific analytical methods besides biomarker staining are required. Studies of the network transformation of lipophilic structures that construct cell components including the endosomal-lysosomal system, a highly dynamic membrane-enclosed vesicular-tubular structure, can provide some indication to facilitate understanding the phenotypic changes of cells after nanoparticle internalization. In regions of cytosol, we found that different organizations of the lipophilic network appeared in cells after pPAuNC internalization as visualized by CLLD staining. To examine lipophilic network remodeling, we introduce a method of single-cell branched lipophilic network analysis (Fig. 7a). First, the CLLD-stained cell images were transformed into black and white images with a supervised threshold and binarization. Then, the connections and branch structure of lipophilic organelles were tagged by the coloring scheme of violet (junction), slab (scarlet), and end-point (blue) for network analysis and classification. In the magnified image of the bPAuNC-treated single-cell state shown in Fig. 7b, the lipophilic networks were preferentially located in the perinuclear/nuclear region and showed aggregated staining patterns. The leak out of the CLLD dye was also confirmed throughout the cell. In contrast, the co-localized regions of pPAuNC and CLLD were generally found in a region anterior to the nucleus. This region is related to the direction of movement and the dot-like staining patterns were retained within a cell. MDA-MB-231 cells migrate in a circular sector shape with a specific direction and they exhibit high MT1-MMP expression in that direction. As verified by Fig. 4, the localized lipophilic networks in the opposite region of the nucleus showed enhanced interaction with MT1-MMP. In addition, the color-tagged skeletonized network was more complex in pPAuNC-treated cells. The image and counts of branch, junction (> 2 neighbors), and slab (= 2 neighbors) voxels indicated the formation of a dense network after pPAuNC internalization (Fig. 7c). Collectively, the results show that differences in the endocytic pathway induce lipophilic structure rearrangements and the transformation of the skeletonized network can demonstrate localized interaction with nanoparticles as well as impaired cellular components.

## Conclusion

Au nanoclusters with stable optical properties have shown promising applicability such as through enhancing the fluorescence properties by controlling the number of Au atoms or recognizing specific molecules by attaching ligands [54, 55]. In this report, we thus synthesized pPAuNC as a photo-stable molecular imaging probe by chemical conjugation of albumin-based gold nanoclusters and peptides. The prepared pPAuNC can efficiently target the MT1-MMP expressed in aggressive cancer cells. The pPAuNC demonstrated stable fluorescent capability even at low pH and was evaluated as a biocompatible agent capable of in vitro imaging. In addition, the MT1-MMP-specific intracellular uptake and targetable imaging property of pPAuNC were confirmed by co-localization analysis against MT1-MMP at a single-cell level. In particular, the location of pPAuNC, which is accumulated in cells after endocytosis, can be confirmed by co-localization analysis with membrane-bound cell organelles composed of lipophilic structures. To confirm the efficacy and potential of nanoparticles specifically, it is important to understand the structural transformation of intracellular organelle networks induced by nanoparticles interacting with other organelles. As presented results, disparate connectivity or branching patterns between organelle networks was identified according to the endocytic pathway of the nanoparticles targeted by the peptide. Moreover, the results of the graphical analysis were consistent when the pixel numbers of junctions, slabs, and end-points were divided by the cell number in the multiple-cell images compared with the network structure image at the single-cell level. Thus, we present the albumin-based photoluminescent nanoparticles as promising photo-stable agents for expanding into functional imaging and therapeutics for cancer treatment alongside RNA therapeutics, anticancer agents, and inhibitors. Furthermore, the intracellular lipophilic network analysis for profiling single cells represents a novel and systematic methodology for the evaluation of the intracellular behavior of nanoparticles in the development of cancer nanomedicines.

## Data Availability

All data generated or analyzed during this study are included in this published article and its additional file.
